# Molecular and Chemical Regulation of the Keap1-Nrf2 Signaling Pathway

**DOI:** 10.3390/molecules190710074

**Published:** 2014-07-10

**Authors:** Young-Sam Keum, Bu Young Choi

**Affiliations:** 1 College of Pharmacy, Dongguk University, 813-4 Siksa-dong, Goyang, Gyeonggi-do 410-820, Korea; 2 Department of Pharmaceutical Science and Engineering, Seowon University, Cheongju, Chungbuk 361-742, Korea

**Keywords:** Keap1, Nrf2, ARE, oncogene, tumor suppressor gene, metabolism

## Abstract

Extracellular and intracellular oxidants or electrophiles are key contributors to the damages in cellular macromolecules, such as DNA, proteins and lipids. Nrf2 is a master transcription factor that modulates a cellular antioxidant response program and plays an important role in the protection against oxidants and electrophiles. Keap1 is a regulator of Nrf2 by serving as a substrate adaptor for Cullin3-dependent E3 ubiquitin ligase. While Nrf2 activation is a feasible strategy for treatment of age-related diseases, aberrant Nrf2 activation also confers a selective growth advantage of tumor cells during chemotherapy or radiotherapy. In the present review, we provide an overview of the Keap1-Nrf2-ARE system, the domain organization of Nrf2 and Keap1, and the regulatory mechanisms of Nrf2 proteolysis by Keap1. We also discuss how Nrf2 prevents tumor promotion, hampers the sensitivity of selected tumors against chemotherapy or radiotherapy, and reprograms the metabolism to facilitate the tumor proliferation. Finally, we illustrate the current status in the development of Nrf2 chemical activators and inhibitors for the use of potential chemopreventive agents and chemotherapeutic adjuvants, respectively.

## 1. Regulation of Cellular Homeostasis by Nrf2 and Keap1

**Figure 1:**
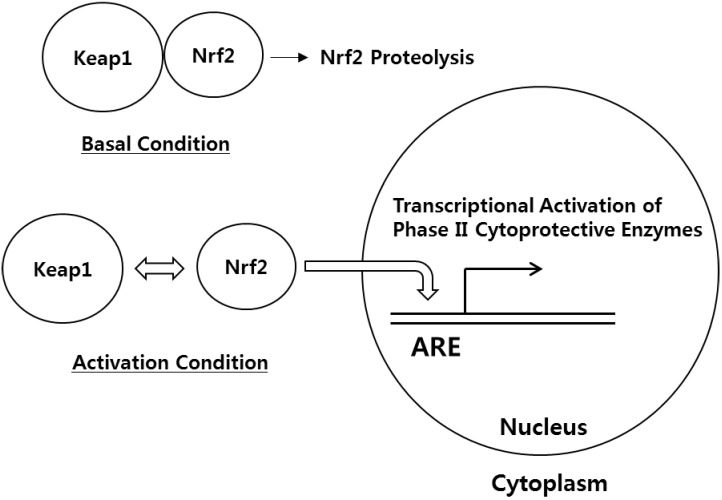
Regulation of ARE-dependent Gene Expression by Keap1 and Nrf2.

Living cells maintain homeostasis by adjusting the intracellular oxidation and reduction levels. However, this intricate balance is constantly challenged by various environmental oxidants and electrophiles, such as pollutants, food additives, ultraviolet, and ionizing radiations [1]. In addition, the generation of intracellular reactive oxygen species (ROS) as by-products during oxidative phosphorylation, including superoxide anion (O_2_^−^), hydroxyl radical (OH^−^), and hydrogen peroxide (H_2_O_2_) contributes to the perturbation of homeostasis [2]. It is well accepted that chronic exposure of human to electrophilic and oxidative stress contributes to a broad range of age-related pathogenesis, such as cancer, cardiovascular, and neurodegenerative diseases [3]. In order to combat against these insults, mammalian cells have developed the elaborate defense mechanisms to confer appropriate adaptive responses [4]. The ubiquitously expressed NF-E2-related factor-2 (Nrf2) is a transcription factor that modulates the basal and inducible expression of phase II cytoprotective enzymes, such as heme oxygenase-1 (HO-1), NAD[P]H:quinone oxidoreductase-1 (NQO1), superoxide dismutase (SOD), glutathione *S*-transferase (GST), and γ-glutamyl cysteine ligase (γ-GCL) [5]. Nrf2 regulates the transcriptional activation of these enzymes by binding to a nucleotide motif sequence existing in 5'-upstream promoter region, e.g., the antioxidant response element (ARE) [6]. ARE contains a consensus sequence 5'-TGACnnnGC-3' and was first identified in the regulatory regions of rat and mouse Gst and Nqo1 genes [7]. Nrf2 is sequestered by the Kelch-like ECH-associated protein 1 (Keap1) in the cytosol and constitutively targeted for poly-ubiquitination under a basal condition. Exposure of cells to electrophilic and oxidative stress, however, releases Nrf2 from Keap1, which enables Nrf2 to translocate to the nucleus and activate a battery of phase II cytoprotective genes by binding to the ARE (Figure 1). In addition to phase II cytoprotective genes, Nrf2 is known to control the transcription of various drug metabolizing enzymes (DMEs), transporters, and cellular reducing equivalents (GSH and NADPH) [8].

## 2. Domain Architecture of Nrf2 and Keap1 and Their Functional Interplay

**Figure 2:**
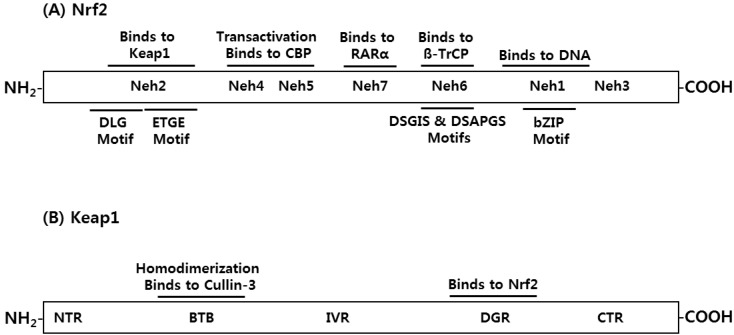
Conserved Domains of Nrf2 and Keap1 Proteins. (**A**) Nrf2 contains seven Neh domains (Neh1-7), in which the Neh1 domain binds to DNA using the bZIP motif and the Neh2 domain interacts with Keap1 using the DLG and ETGE motifs. The Neh4 and Neh5 domains are required for gene transactivation. The Neh6 domain binds to β-TrCP using DSGIS and DSAPGS motifs. The Neh7 domain binds to RARα and suppresses the Nrf2 activity; (**B**) Keap1 contains five different domains (NTR, BTB, IVR, DGR and CTR), in which the BTB domain forms a homodimer for binding to Cullin-3 and the DGR domain forms a six-blade propeller with 6x Kelch motifs for the interaction with Nrf2.

In addition to the transcriptional activation of phase II cytoprotective enzymes, Nrf2 is linked to a number of other cellular processes, such as differentiation, proliferation, cell growth, apoptosis and hematopoiesis by closely interacting with diverse intracellular regulatory proteins [9]. Nrf2 is a basic leucine zipper (bZIP) transcription factor that contains a Cap’n’Collar (CNC) structure and most transcription factors that harbor the CNC structure are transcriptional activators [10]. Domain analysis of Nrf2 has illustrated that Nrf2 is composed of seven conserved Nrf2-ECH homology (Neh) domains (Figure 2A). The Neh1 domain contains the bZIP motif, which enables Nrf2 to bind to the ARE sequence. In addition, this domain can interact with UbcM2, E2 ubiquitin conjugating enzyme, to regulate the Nrf2 protein stability [11]. The Neh2 domain, located in the most N-terminal region, acts as a negative regulatory domain by binding to Keap1 [12]. The Neh3 domain, located in the most C-terminal region, is known to assist Nrf2 transactivation through the interaction with chromatin remodeling protein CHD6 [13], while the tandem Neh4 and Neh5 domains are critical for Nrf2-mediated ARE transactivation by binding to a transcriptional coactivator, e.g., CBP [14]. The redox-insensitive Neh6 domain possesses DSGIS and DSAPGS motifs and provides a phosphorylation-dependent binding platform for β-TrCP [15]. Finally, Wang *et al.* have recently identified the novel domain in Nrf2, e.g., the Neh7 domain, that interacts with the retinoic acid receptor α (RARα) and represses Nrf2 target gene expression [16].

**Figure 3:**
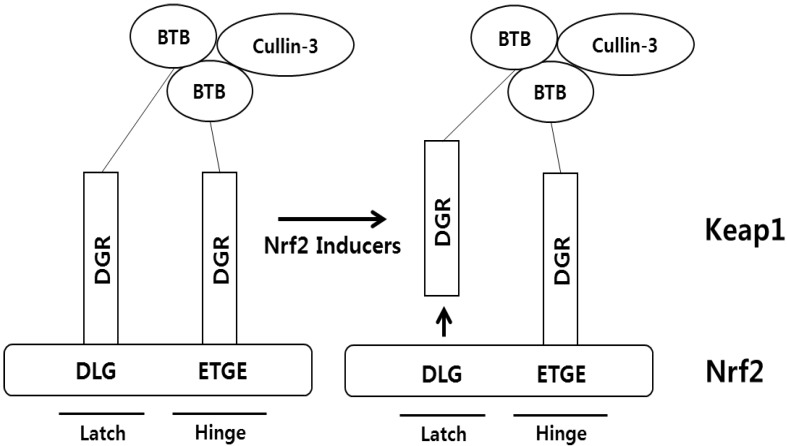
The “Hinge and Latch” Hypothesis. Under basal conditions, Keap1 forms a homodimer and associates with Cullin-3 protein. At the same time, the DGR domains of two Keap1 bind to the DLG (latch) and the ETGE (hinge) domains in a single Nrf2. In response to Nrf2 inducers, the DLG motif in Nrf2, but not ETGE motif in Nrf2, is released from the DGR domain in Keap1.

Keap1 is a cytosolic protein that inhibits the ARE-dependent gene expression by binding to the Neh2 domain of Nrf2. In fact, Keap1 was initially identified by yeast 2-hybrid assay, using the Neh2 domain of Nrf2 as bait [17]. Keap1 consists of 5 different domains: an amino-terminal region (NTR), a Broad complex, Tramtrack and Bric a brac domain (BTB), an intervening region (IVR), six Kelch/double glycine repeats (DGR), and a carboxyl terminal region (CTR) (Figure 2B). The cytoplasmic location of Keap1 can be explained, at least in part, by its binding ability to a cytoplasmic actin or myosin VIIa through the DGR domain [18]. Keap1 also employs the DGR regions to recognize two primary sequences, e.g., the ETGE and DLG motifs, existing in the Neh2 domain of Nrf2 protein by forming a six-bladed propeller [19]. In addition, two interesting features underlying the interaction between Nrf2 and Keap1 exists. First, Keap1 can dimerize with each other, using the BTB domain to interact with Cullin-3. Second, two Keap1 proteins bind to a single Nrf2 protein at a ratio of 2:1 [20], in which the overlapping ETGE and DLG motifs in Nrf2 bind to two Keap1 proteins with a differential affinity: a single Keap1 strongly binds to the ETGE motif of Nrf2 (Ka = 20 × 10^7^ M^−1^) and, at the same time, another Keap1 interacts with the DLG motif with a weak affinity (Ka = 0.1 × 10^7^ M^−1^) [21]. Based on these observations, so called the “hinge and latch” hypothesis was proposed to explain the regulatory mechanism of Nrf2 by Keap1 (Figure 3), in which the “hinge” mediates a high-affinity interaction between the ETGE motif of Nrf2 and Keap1 and this interaction is unaffected by stress inducers, whereas the “latch” mediates displacement of the DLG motif of Nrf2 from Keap1 in response to treatment of Nrf2 inducers [22].

The cellular Nrf2 protein level is mediated, largely in part, by the ubiquitin-mediated proteolysis [23]. Ubiquitin is a 76 amino-acid protein whose main function is to mark proteins for degradation. The ubiquitin-mediated proteolysis requires a cascade of three enzymes: E1 (ubiquitin-activating), E2 (ubiquitin-conjugating), and E3 (ubiquitin-ligase) enzymes [24]. The E3 ubiquitin ligases contain either the homologous to E6-associated protein (E6-AP) COOH-terminus (HECT) domain or the really interesting new gene (RING) finger domain [25]. While the HECT-type E3 ubiquitin ligases display a catalytic activity by itself, the RING finger-type E3 ubiquitin ligase promotes the poly-ubiquitination of substrates by positioning substrates in a close proximity to the activated E2 enzymes (ROC1 or ROC2) through molecular assembly by Cullin proteins [26]. Cullins (Culs) consist of seven different isotypes in human (Cul1, 2, 3, 4A, 4B, 5, and 7) and serve as scaffold proteins to assemble the Cullin-RING E3 ubiquitin ligases [27]. Since Keap1 possesses the BTB domain, Keap1 behaves as an adaptor module for Cul3-type E3 ubiquitin ligase complex, contributing to a constant poly-ubiquitination of Nrf2 in a stretch of lysine (K) residues that exist in the ETGE-DLG intervening region of Nrf2 [28]. Additionally, recent studies have illustrated that the β-transducin repeat-containing protein (β-TrCP), an adaptor protein for the Cul1-Skp1-ROC1 E3 ubiquitin ligase complex, also targets Nrf2 for poly-ubiquitination and proteolysis, following GSK3β-mediated phosphorylation upon the DSGIS motif existing in the Neh6 domain of Nrf2 (Figure 2A) [29]. A follow-up study illustrated that the Neh6 domain possesses an additional recognition motif for β-TrCP, e.g., DSAPGS motif and that the binding of β-TrCP to this motif is not influenced by GSK activity (Figure 2A) [30]. At present, the detailed mechanisms how Keap1 and β-TrCP discriminate Nrf2 for poly-ubiquitination are largely unclear.

Dinkova-Kostova and colleagues have recently attempted to visualize the molecular interaction between EGFP-Nrf2 and Keap1-mCherry in cells by using a quantitative fluorescence resonance energy transfer (FRET) technique, coupled with fluorescence lifetime imaging microscopy (FLIM). FRET is a physical mechanism that describes the energy transfer between two light-sensitive molecules and frequently used to interrogate the proximity between two proteins in cells [31]. Based on the FLIM-FRET observations, they found that the Keap1/Nrf2 complex exists in two different conformations: (1) an open conformation in which only the high affinity “ETGE” motif, but not the low affinity “DLG” motif in Nrf2 is bound to Keap1 dimers and (2) a closed conformation in which both the “DLG” and “ETGE” motifs are bound to Keap1 dimers [32]. Contrary to the “hinge and latch hypothesis”, they observed that treatment of Nrf2 inducers [sulforaphane and sulfoxythiocarbamate (STCA)] increased the abundance of closed conformation in cells. Based on these data, authors proposed a new model, related to the Keap1/Nrf2 interaction and referred it to as “conformational cycling model” or “cyclic sequential attachment and regeneration model” According to this model, Nrf2 inducers function to promote Nrf2 stabilization by facilitating the formation of closed conformation, but not open conformation, of the Keap1-Nrf2 complex [33]. Although the Keap1-Nrf2 complex exists in the closed conformation, the direct binding of Nrf2 inducers to Keap1 seems to uncouple Nrf2 for targeted degradation possibly due to Keap1 conformational changes. As a result, Nrf2 is not released from Keap1 and, therefore, the free Keap1 dimer is not regenerated in cells. Because the newly synthesized Nrf2 is unable to bind to Keap1 due to the lack of free Keap1 dimers, Nrf2 is free to translocate into the nucleus and activates ARE-dependent gene expression.

## 3. Nrf2: Tumor Suppressor Gene or Oncogene?

Nrf2 knock-out mice are highly susceptible for drug-induced toxicity and oxidative stress-related diseases, such as cardiovascular diseases, acute lung injury, diabetes, and inflammation [34]. In addition, there are a number of studies illustrating that Nrf2 knock-out mice are more prone to develop chemically-induced tumors, compared with their wild-type littermates [35,36,37]. Similar to this observation, a conditional Keap1 disruption in mice reduced the level of cell damages, caused by oxidative stress and prevented the onset and exacerbation of stress-induced diseases [38]. Together, these results indicate that Nrf2 is a critical oxidative stress regulator. In addition, the attempts to elucidate novel Nrf2 or Keap1 binding proteins and their functions in cells have illustrated that Nrf2 is a tumor suppressor, important for the inhibition of tumor initiation. It is well established that, in response to mild genotoxic or oxidative stresses, p53 activates the transcriptional expression of p21, which in turn causes the cell cycle arrest or senescence at the G1/S boundary in normal cells by binding to G1/S cyclin-dependent kinases [39]. Interestingly, Zhang and colleagues have identified that p21 protein can bind to the DLG motif in Nrf2 and increase the Nrf2 stability by displacing Nrf2 from Keap1-dependent poly-ubiquitination [40]. This result implies that the anti-tumorigenic effects of Nrf2 can be attributed, at least in part, to its interaction with the p53-p21 axis and the resulting cell cycle arrest that favors the DNA damage repair [41]. The other Keap1 binding proteins, such as the Wilms tumor gene on the X chromosome (WTX) [42], PALB2/FANCN [43], and dipeptidyl peptidase 3 (DPP3) [44] also contain the ETGE motif in common and can promote the Nrf2 protein stability by competitively binding to Keap1. In particular, WTX and PALB2 are considered as *bona fide* tumor suppressors [45]. Therefore, it can be concluded that WTX and PALB2-mediated tumor suppression can be ascribed, at least in part, to maintaining an increased cellular Nrf2 level.

Although it seems counter-intuitive, Nrf2 activity is highly up-regulated in various types of tumors and the prognosis of patients with tumors expressing high levels of Nrf2 is poor [46]. In addition, somatic gain of function mutations in Nrf2 gene were identified in tumors and these mutations are clustered in the proximity of the DLG and ETGE motifs, suggesting that the hinge and latch hypothesis holds in the clinical setting [47,48,49]. Moreover, somatic heterozygous loss of function mutations in Keap1 were also identified in various tumor tissues [46]. Using a genetically-engineered mouse model, Suzuki *et al.* have demonstrated that Keap1 heterozygous mutation is sufficient to cause an increased Nrf2 activity [50]. These observations illustrate that cancer cells can hijack the Keap1/Nrf2 pathway to keep their intracellular ROS burden within a range, thereby permitting a proper growth and survival for tumor cells. Supporting this view, DeNicola *et al.* have reported that the activation of oncogenes, such as K-Ras, B-Raf, and c-Myc stimulates Nrf2 transcription via MAPK pathway and this event contributes to oncogene-stimulated cell proliferation *in vivo* by maintaining the intracellular ROS level below a threshold [51]. However, it appears that a simple accumulation of Nrf2 is not sufficient to incur a spontaneous tumor development [52]. Therefore, it would be the most appropriate to interpret that Nrf2 exhibits tumor-suppressive functions in normal cells, e.g., “a good side of Nrf2”, but cancer cells hijack the survival property of Keap1-Nrf2 pathway to confer a selective advantage to sustain their proliferation and stress resistance, e.g., “a bad side of Nrf2”. This conjecture is well supported by a recent observation that Nrf2 prevents initiation, but accelerates progression of tumors during urethane-induced lung carcinogenesis in mice [53].

A growing amount of evidence indicates that Nrf2 can rewire metabolic programs to promote cell growth and proliferation in cancer [54]. Mitsuishi *et al.* have demonstrated that Nrf2 can redirect glucose and glutamine into anabolic pathways [55]. In addition, Nrf2 regulates the expression of glucose-6-phosphoate dehydrogenase (G6PD), 6-phosphogluconate dehydrogenase (PGD), malic enzyme-1 (ME1), and isocitrate dehydrogenase-1 (IDH1), all of which can convert oxidized NADP^+^ into its reduced form, NADPH [56]. Because NADPH provides a reducing power in cells and is required for the biosynthesis of lipids and nucleotides, it is possible to assume that Nrf2 might contribute to an increased cell proliferation in tumors by providing a sufficient amount of NADPH. Another interesting target gene of Nrf2 is the pyruvate kinase (PK), a critical and final glycolytic enzyme that irreversibly converts phosphoenolpyruvate (PEP) into pyruvate. Interestingly, Nrf2 was shown not to increase, but rather decrease the PK expression [57]. Although the detailed mechanism is unclear yet, it is possible to surmise that a decreased PK expression would have promoted the accumulation of glycolytic intermediates, which were in turn diverted towards the pentose phosphate pathway. In addition to the regulatory of Nrf2 in metabolic genes, fumarate, a metabolite in the tricarboxylicacid (TCA) cycle, was shown to form a direct adduct with Keap1 and activates the Nrf2-dependent gene expression in the hereditary form of type 2 papillary renal carcinoma [58]. Likewise, an accumulation of fumarate modified selected cysteine residues in Keap1 and abrogated the function of Keap1 to promote Nrf2 proteolysis and it led to an increase in the renal cyst formation of fumarate hydratase (FH)-deficient mice [59]. These observations fit well with the postulated tumor suppressor functions of certain Krebs cycle enzymes, such as isocitrate dehydrogenase (IDH), succinate dehydrogenase (SD) and fumarate hydratase (FH) [60]. Further detailed mechanistic studies, addressing how accumulated metabolites in the intermediary metabolism cooperate with the Keap1/Nrf2 pathway to assist the tumor development will be an interesting theme of future investigations.

## 4. Development of Chemical Nrf2 Inducers and Inhibitors

Chemoprevention has emerged as an important research discipline in recent years [61]. Natural chemopreventive agents, such as curcumin, sulforaphane are strong chemical inducers of ARE-dependent phase II cytoprotective enzymes [62]. Accumulating evidence indicates that ARE-dependent gene activation by chemopreventive compounds is mediated by modulating the activities of intracellular signaling kinase pathways. For example, the mitogen-activated protein kinases (MAPKs) are implicated in the regulation of ARE-dependent gene expression, in which ERK and JNK are positive regulators while p38 MAPK is a negative regulator in response to treatment of chemopreventive agents [63]. On the other hand, there exist numerous intracellular kinases that are involved in ARE-dependent gene regulation as well, such as phosphatidylinositol 3'-kinase (PI3K), PKR-like endoplasmic reticulum kinase (PERK), protein kinase C (PKC), Fyn kinase, and glycogen synthase kinase-3β (GSK3β) [64]. However, the detailed molecular mechanisms underlying how Nrf2 phosphorylations contribute to ARE-dependent gene expression are largely unclear except for PKCδ and Fyn kinases. Niture *et al.* have reported that PKCδ activation results in phosphorylation of Nrf2 at Ser40 and it contributes to the escape or release of Nrf2 from Keap1 [65]. On the other hand, Jain *et al.* have reported that activation of GSK3β directly phosphorylates Fyn, leading to a nuclear localization of Fyn and Nrf2 phosphorylation at Tyr568 [66]. Interestingly, they have observed that Fyn-mediated Nrf2 phosphorylation at Tyr568 suppressed Nrf2 transactivation by facilitating a nuclear export of Nrf2.

**Scheme 1:**
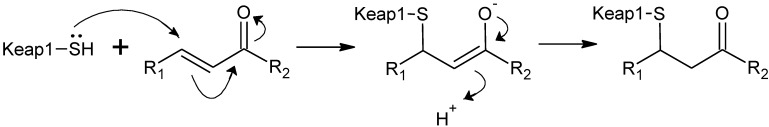
Adduct Formation of Keap1 with Michael Acceptor.

In an insightful review, Magesh *et al.* have classified around 90 natural or synthetic Nrf2 chemical activators as follows: (1) Michael acceptors; (2) oxidizable phenols and quinones; (3) isothiocyanates and sulfoxythiocarbamates; (4) dithiolethiones and diallyl sulfides; (5) trivalent arsenicals; (6) vicinal dimercaptans; (7) selenium-based compounds; (8) polyenes; (9) hydroxyperoxides; (10) heavy metals and metal complexes; and (11) miscellaneous inducers [67]. While the biochemical mechanisms how these chemicals induce ARE-dependent gene expression are largely elusive, it is known that Michael acceptors promote Nrf2-dependent ARE-gene expression by forming a covalent adduct with the multiple sulfhydryl groups of cysteines in Keap1, an interesting hypothesis referred to as the “cysteine code hypothesis”. According to this hypothesis, Keap1, but not Nrf2, is a stress sensor. Michael acceptors are directly conjugated to cysteine (Cys) residues in Keap1 (Scheme 1) and these modifications lead to a conformational change of Keap1, resulting in the release of Nrf2 from the low affinity binding site (the latch) from poly-ubiquitination [68]. Among more than 20 cysteine residues found in Keap1, Cys-151. Cys-273, and Cys-288 were identified to serve as critical residues of stress sensors *in vivo* [69]. In addition, Hayes and colleagues have demonstrated using mass spectrometry that endogenous or exogenous chemical inducers of Nrf2 modified different Cys residues of Keap1 in cells [70]. Because a large number of Michael acceptors are found in naturally-occurring chemopreventive agents [curcumin, caffeic acid phenethyl ester (CAPE), flavonoids and sesquiterpenes as examples] all of which possess olefins conjugated with electron-withdrawing carbonyl groups in common, finding out whether and which Cys residues in Keap1 these natural compounds directly modify to exhibit Nrf2-dependent phase II cytoprotective gene activation is necessary to better clarify their chemopreventive mechanisms.

**Figure 4:**
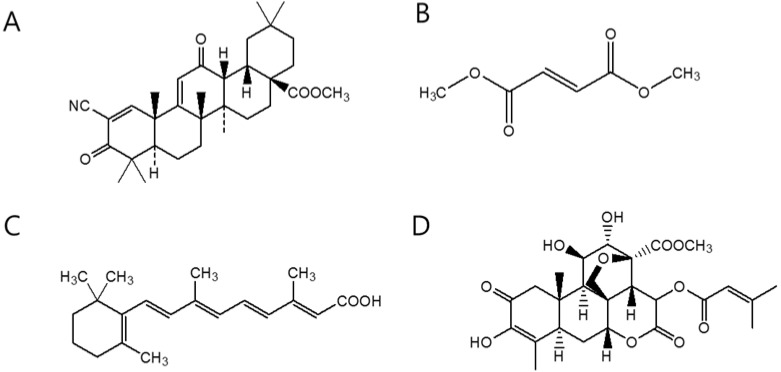
Chemical Structures of (**A**) CDDO-Me; (**B**) DMF; (**C**) ATRA; and (**D**) brusatol.

Considering that oxidative stress and inflammation are intimately correlated with each other, pharmaceutical companies have recently attempted to develop Nrf2 chemical activators as antioxidant inflammation modulators (AIMs). One of the most promising Nrf2 inducers was the methyl ester derivative of the synthetic triterpenoid, 2-cyano-3,12-dioxooleana-1,9(11)-dien-28-oic acid (CDDO-Me, Figure 4A). Because CDDO-Me induces Nrf2 at low nanomolar concentrations [71], it underwent development as a promising AIM under the generic name, bardoxolone methyl for treatment of advanced chronic kidney disease (CKD) and type 2 diabetes mellitus [72]. Unfortunately, the clinical trial of bardoxolone methyl was terminated in the phase III in October 2012 for safety concerns due to excess serious adverse events and mortality. Dimethyl fumarate (DMF, Figure 4B) is another synthetic Nrf2 activator that has been developed for potential AIM. Scannevin *et al.* have demonstrated that treatment of primary cultured central nervous system (CNS) cells or animals with DMF resulted in an increased nuclear level of Nrf2 and Phase II cytoprotective genes, thereby rendering a significant protection against oxidative challenges [73]. More importantly, an oral preparation of DMF successfully reduced the annual relapse rate in patients with multiple sclerosis (MS) in the phase III clinical trial [74] and the Food and Drug Administration (FDA) finally approved DMF for treatment of MS in March 2013 under the trade name of Tecfidera. Whether DMF possesses chemopreventive activities is unknown at present.

On the other hand, the development of Nrf2 chemical inhibitors started to garner a significant interest in recent years due to the realization that a high level of Nrf2 is implicated in the chemoresistance and radioresistance [75]. Wolf and colleagues have previously reported that all-*trans* retinoic acid (ATRA, Figure 4C) is a chemical inhibitor of Nrf2. As such, they have demonstrated that treatment of ATRA or other retinoic acid receptor-α (RARα) agonists reduced the ability of Nrf2 binding to the ARE and suppressed the induction of ARE-driven genes by butylated hydroxyanisole (BHA) and *tert*-butyl hydroxyquinone (tBHQ). [76]. It is well known that retinoids, including ATRA exhibit significant chemopreventive effects against head and neck cancer [77]. The effects of retinoids are mediated by specific nuclear receptors, e.g., retinoic acid receptors (RAR-α, -β, and -γ) and retinoid X receptors (RXR-α, -β, and -γ), in which RAR and RXR form a heterodimer and exerts their biological effects by binding to retinoic acid response elements (RAREs) in the genome [78]. In addition to RARα, other nuclear receptors, such as peroxisome proliferator-activated receptor-γ (PPARγ), estrogen receptor-α (ERα), estrogen-related receptor-ß (ERRβ), and glucocorticoid receptor (GR) are reported to physically interact with Nrf2 and repress Nrf2-dependent gene expression [79], raising an intriguing possibility that Nrf2 can be implicated in hormone-related diseases. In addition to ATRA, several other natural chemicals have been reported to exert inhibitory effects on the Nrf2-ARE pathway. Zhang and colleagues have demonstrated that brusatol (Figure 4D), purified from the extract of plant Brucea javanica suppressed Nrf2-dependent gene expression and increased the sensitivity of cisplatin-mediated apoptosis of cancer cells *in vitro* and *in vivo* [80]. Similarly, recent studies have illustrated that several natural chemicals, such as luteolin [81], procyanidin [82], apigenin [83], and chrysin [84] can suppress the Nrf2 activity. The molecular mechanisms by which these natural compounds exhibit inhibitory effects on the Nrf2-ARE pathway are largely unclear. However, it should be noted that there exist other literatures illustrating that these compounds can act as Nrf2 activators in different experiment settings. Therefore, further studies will be necessary to establish the specificity and accurate mechanisms of action of these chemicals, if they are intended for development of chemotherapeutic adjuvants in the future.

## 5. Concluding Remarks

By now, we have discussed the regulatory mechanisms of the Keap1-Nrf2-ARE pathway. However, a growing evidence indicates that the regulation of the Keap1-Nrf2-ARE is more complex than thought and other molecular mechanisms are also involved. Among them, we believe that the epigenetic regulation of Nrf2 [85] and Keap1 [86] will particularly an interesting theme of future studies. Because Nrf2 exhibits protective effects against oxidants and electrophiles in normal cells but sustained Nrf2 activation favor the growth of tumors, it is possible to speculate that differential epigenetic regulation of Nrf2 and/or Keap1 might exist in normal and cancer cells. In addition, we are now beginning to appreciate that Nrf2 plays an important role in the regulation of hormonal outcomes and metabolism. Therefore, the implication of Nrf2 in hormonal and metabolic diseases warrants further investigations considering that the Keap1-Nrf2-ARE pathway is now moving into the realm of drug development.
